# Penalized multivariate linear mixed model for longitudinal genome-wide association studies

**DOI:** 10.1186/1753-6561-8-S1-S73

**Published:** 2014-06-17

**Authors:** Jin Liu, Jian Huang, Shuangge Ma

**Affiliations:** 1 School of Public Health, University of Illinois at Chicago, 1601 W. Taylor Street, Chicago, IL 60612, USA; 2 Department of Statistics & Actuarial Science, Department of Biostatistics, University of Iowa, 241 Schaeffer Hall, Iowa City, IA 52242, USA; 3 School of Public Health, Yale University, 60 College Street, New Haven, CT 06520, USA; 4 VA Cooperative Studies Program Coordinating Center, West Haven, CT 06516, USA

## Abstract

We consider analysis of Genetic Analysis Workshop 18 data, which involves multiple longitudinal traits and dense genome-wide single-nucleotide polymorphism (SNP) markers. We use a multivariate linear mixed model to account for the covariance of random effects and multivariate residuals. We divide the SNPs into groups according to the genes they belong to and score them using weighted sum statistics. We propose a penalized approach for genetic variant selection at the gene level. The overall modeling and penalized selection method is referred to as the penalized multivariate linear mixed model. Cross-validation is used for tuning parameter selection. A resampling approach is adopted to evaluate the relative stability of the identified genes. Application to the Genetic Analysis Workshop 18 data shows that the proposed approach can effectively select markers associated with phenotypes at gene level.

## Background

The Genetic Analysis Workshop 18 (GAW18) data consists of multiple longitudinal traits and dense genome-wide single-nucleotide polymorphism (SNP) markers. A commonly used approach for identifying markers associated with traits is to conduct single-variant analysis and then adjust for multiple comparisons on each trait. However, for complex polygenic traits, single-variant analysis methods may not be appropriate as they fail to take into account the accumulated and/or joint effects of multiple genetic variants on the traits. In addition, analyzing each trait separately does not take into account the correlation among traits, and thus can be ineffective. To overcome these limitations, we developed a joint analysis approach referred to as the penalized multivariate linear mixed model (PMLMM). This approach takes into account covariance of both random effects and residuals and uses a group minimax concave penalty (MCP) approach [1] for variant selection at the gene level. A resampling approach is adopted to evaluate the relative stability of the identified genes. Our analysis of the GAW18 data indicates that the proposed approach can effectively select markers associated with multiple traits at the gene level.

## Methods

Consider a genetic association study with longitudinal measurements on *N *subjects, *p *genetic variants, and *q *environmental exposure covariates. Here a genetic variant can be a single SNP marker or a score representing a group of SNPs. For subject *i*, suppose that there are $n_{i}$ longitudinal measurements on *m *traits. Let $Y_{i}$ be the $n_{i}\times m$ trait matrix for subject *i*. Let *Y *be the n×m trait matrix for all the *N *subjects, where $n=\sum_{i=1}^{N}n_{i}$. The transpose of *Y *is $Y\prime =\left(Y_{1}^{\prime },\cdot \cdot \cdot ,Y_{N}^{\prime }\right)$. Let $X_{i}$ be the $n_{i}\times p$ matrix consisting of the genetic variant scores of subject *i*. Let $Z_{i}$ be the $n_{i}\times q$ covariate matrix. We center all the measurements to have sample means equal to zero. When m=1, this setting simplifies to that in Schelldorfer et al [2].

Consider the multivariate linear mixed model

(1) $$Y_{i}=X_{i}B+Z_{i}C_{i}+E_{i},\;i=1,...,N,$$

where *B *is a p×m matrix representing the effects of *p *genetic variants on *m *traits, and $C_{i}$ is a q×m matrix representing the subject specific effects of the covariates $Z_{i}$ for the *i*^th ^subject. We treat $C_{i}$ as random effects. Assume that (a) $E_{i}\sim \text{M}{\text{N}}_{n_{i}\times m}\left(0,\underset{1}{\sum },I_{n_{i}}\right)$, that is, $E_{i}$ is row-independent with column covariance matrix $\underset{1}{\sum }$, and each $E_{i}$ is independent for i=1,...,N; (b) $C_{i}\sim \text{M}{\text{N}}_{q\times m}\left(0,\underset{2}{\sum },D\right)$, where $\underset{2}{\sum }$ is the column covariance matrix and *D *is the row covariance matrix, and each $C_{i}$ is independent for i=1,...,N; (c) each $E_{i}$ and $C_{i}$ is independent; and (d) $\underset{1}{\sum }=\underset{2}{\sum }=\sum$.

Then $Z_{i}C_{i}+E_{i}\sim \text{M}{\text{N}}_{n_{i}\times m}\left(0,\sum ,Z_{i}D\overset{\prime }{\underset{i}{Z}}+I_{n_{i}}\right)$ and $Y\sim \text{M}{\text{N}}_{n\times m}\left(XB,\sum ,V\right)$ where $V=Diag\left(V_{1},...,V_{N}\right)$ and $V_{i}=Z_{i}DZ_{i}^{\prime }+{\text{I}}_{n_{i}}$, where ${\text{I}}_{n_{i}}$ is an $n_{i}\times n_{i}$ identity matrix. A more detailed description of this model can be found in Liu et al [3].

From Dawid [4], the negative log-likelihood function is:

(2) $$-\ell \left(B,V,\sum \right)=\text{constant}+\frac{n}{2}\text{log}|\sum |+\frac{m}{2}\text{log}|V|+\frac{1}{2}\text{tr}\left(\overset{-1}{\sum }\left(y\text{ - }\;XB\right)\prime V^{-1}\left(y\text{ - }\;XB\right)\right)$$

Hastie et al. [5] suggest using $\hat{\sum }=y\prime y/n$ for estimating *∑*. We estimate *D *by using the estimates from *m *univariate linear mixed models and subsequently get the estimate $\hat{V}$ of *V *as ${\hat{V}}_{i}=Z_{i}\hat{D}Z_{i}^{\prime }+{\text{I}}_{n_{i}}$. Given $\hat{\sum }$ and $\hat{V}$, we can transform the negative likelihood function into a weighted least squares criterion for estimating *B*, which is $\text{tr}\left({\hat{\sum }}^{-1}\left(y\text{ - }\;XB\right)\prime {\hat{V}}^{-1}\left(y\text{ - }\;XB\right)\right)$. For variant selection, we adopt the group MCP approach [6]. The overall penalized objective function is

(3) $$\text{Q}\left(B\right)=\text{tr}\left({\hat{\sum }}^{-1}\left(y\text{ - }\;XB\right)\prime {\hat{V}}^{-1}\left(y\text{ - }\;XB\right)\right)+\sum_{j=1}^{p}\rho \left(||B_{j}|{|}_{2};\lambda ,\gamma \right),$$

where $B_{j}$ is the *j*^th ^row of B and $\rho \left(t;\lambda ,\gamma \right)=\lambda \int_{0}^{\left|t\right|}{\left(1-x/\left(\gamma \lambda \right)\right)}_{+}dx$ is the MCP with tuning parameter *λ *and regularization parameter *γ*[7].

For computation, we use a group coordinate descent algorithm [1]. The group MCP involves a regularization parameter and a tuning parameter. Generally speaking, smaller values of *γ *are better at retaining the unbiasedness of the MCP penalty for large coefficients, but they have the risk of creating objective functions that have problems with nonconvexity [8], are difficult to optimize, and yield solutions that are discontinuous with respect to *λ*. Simulation studies by Breheny and Huang [8] suggest that γ=6 is a reasonable choice. Therefore, we fix it to be 6 in our analyses. We search for the optimal value of *λ *using 5-fold cross-validation.

## Results

The GAW18 data set consists of dense genome-wide markers with longitudinal measurements on systolic and diastolic blood pressure (SBP and DBP) and other covariates. Other measurements include gender, age, year of examination, use of antihypertensive medications, and tobacco smoking at up to 4 time points. In this study, we analyze the 157 unrelated individuals using SBP and DBP as traits and other medical and demographic covariates as random effects. Gene annotations for SNP data are obtained from http://www.scandb.org. SNPs in each gene are scored using weighted sum statistics to generate gene-level measurements [9]. After quality control, we have the genetic scores of 10,400 genes for further analysis. SBP, DBP, and genetic scores are standardized to have zero means and unit variances. This procedure removes the estimation of intercepts and makes the genes comparable.

We apply the proposed PMLMM to identify genetic variants that are associated with both SBP and DBP at the gene level. As a benchmark, we also analyze each trait separately using a penalized linear mixed model (PLMM) approach. Table 1 shows the genes identified using PMLMM. Table 2 summarizes the overlaps of genes selected using the different approaches. Although there is overlap, PMLMM and PLMM identify significantly different sets of genes. We evaluate the relative stability of identification of each gene using a resampling approach and calculate the observed occurrence index (OOI) [10]. A larger value of OOI indicates that the corresponding identified gene is more stably identified. Table 1 also shows OOI results. The identified genes have reasonably high OOIs.

**Table 1:** Genes identified by PMLMM: estimates for SBP and DBP, and OOI

Gene	SBP	DBP	OOI	Gene	SBP	DBP	OOI
*MMEL1*	0.002	−0.002	0.333	*TMEM41B*	0.027	0.033	0.403
*CD52*	0.085	0.060	0.697	*ARNTL*	0.024	0.006	0.247
*DPH2*	0.071	−0.032	0.323	*SPTY2D1*	0.025	−0.007	0.507
*C8A*	0.018	0.032	0.563	*CHST1*	−0.008	−0.031	0.540
*DNAJB4*	−0.028	−0.022	0.333	*MRE11A*	−0.042	−0.007	0.623
*HS2ST1*	0.002	0.006	0.307	*ENOX1*	−0.068	−0.032	0.647
*PROK1*	0.006	0.010	0.373	*LOC100132760*	−0.041	0.048	0.693
*THBS3*	−0.004	0.001	0.337	*SPRY2*	−0.004	−0.005	0.297
*C1orf182*	2E-04	0.045	0.490	*GABRG3*	−0.027	0.006	0.573
*TGFBR2*	−0.033	−0.030	0.627	*THBS1*	0.023	0.028	0.353
*LOC100129194*	0.005	0.011	0.217	*CSPG4*	0.098	−0.012	0.880
*LMOD3*	−0.013	−0.028	0.493	*C15orf27*	0.047	−0.014	0.490
*LOC653712*	0.017	0.001	0.450	*LOC100128570*	0.026	0.019	0.283
*LAMP3*	0.034	0.008	0.627	*HOMER2*	0.024	0.013	0.250
*EIF2B5*	−0.014	−0.014	0.417	*ADAMTS17*	0.002	0.002	0.270
*EHHADH*	0.003	−0.052	0.677	*SLC16A11*	0.015	−0.004	0.170
*SFRS12*	0.005	0.007	0.290	*ALDH3A1*	0.002	0.021	0.657
*C5orf32*	0.019	−0.029	0.560	*FLJ44815*	0.019	−0.005	0.210
*ZNF346*	0.001	−0.024	0.553	*TANC2*	0.003	−0.001	0.187
*LOC100128901*	−0.069	0.006	0.627	*PDE6G*	−0.056	−0.012	0.377
*OGDH*	0.123	0.038	0.777	*C19orf6*	0.013	0.048	0.577
*NSUN5*	0.035	−0.014	0.660	*TMEM146*	−0.001	−0.063	0.550
*PPP1R3A*	−0.034	−0.041	0.453	*STX10*	4E-04	0.015	0.333
*MEST*	−2E-04	−3E-04	0.197	*RLN3*	0.024	−0.034	0.603
*NOM1*	0.029	−0.001	0.630	*CYP4F11*	0.018	0.005	0.127
*FLJ41200*	0.013	0.005	0.333	*LOC728326*	0.048	−0.040	0.547
*LRRC19*	−0.015	−0.040	0.480	*ZNF585A*	0.028	0.082	0.720
*CCIN*	0.006	−0.004	0.437	*SUPT5H*	0.020	−0.014	0.233
*LOC100130911*	0.004	0.024	0.467	*FLJ10490*	0.004	2E-04	0.327
*PTCH1*	0.004	−1E-04	0.300	*ZNF331*	0.027	0.003	0.330
*DFNB31*	0.057	−0.008	0.710	*BACE2*	−0.116	−0.074	0.940
*OR52D1*	0.023	−0.017	0.490	*KRTAP10-12*	0.002	0.003	0.233

**Table 2:** Overlap of selected genes between PMLMM and PLMM

	PMLMM	PLMM*	PLMM†
PMLMM	64	24	16
PLMM^1^		40	0
PLMM^2^			29

*PLMM on SBP.

†PLMM on DBP.

## Discussion

In this study, we analyze the GAW18 data and develop a PMLMM approach. A multivariate linear mixed model is used to model variance components among traits and longitudinal measurements. A penalization approach is adopted for variant selection. In the estimation procedure, it can be considered heuristic to use $\hat{\sum }$ and $\hat{V}$ as proposed. Assumptions (a) to (c) are standard in mixed models, but the assumption that $\underset{1}{\sum }=\underset{2}{\sum }$ may be restrictive. Because our study is to identify multitrait-associated markers at the gene level, the restriction on variance components does not affect the selection result significantly. We are currently developing a similar approach to update variance components with more relaxed assumptions on $\underset{1}{\sum }$ and $\underset{2}{\sum }$. An iterative algorithm can be implemented to solve for *B*, *∑*, and *V*. In variant selection, our method is designed to search for genes associated with all the traits considered. When different sets of genetic variants are suspected to be associated with different phenotypes, the sparse group penalization approach [11] can be applied.

## Conclusions

We have presented a penalized multivariate linear mixed model (PMLMM) for detecting pleiotropic genetic associations among multiple traits in the presence of pedigree structures. The proposed approach combines the advantages of mixed models that allow for elegant correction for pedigree-based family data and integrative analysis of multiple traits. Compared with PLMM which considers one trait at a time, the proposed PMLMM can achieve better performance when the pleiotropic effect is appropriately modeled.

## Competing interests

The authors declare that they have no competing interests.

## Authors' contributions

All authors were involved in study design. JL conducted the numerical work. All authors were involved in manuscript preparation, and read and approved the final manuscript.
